# RAD50 deficiency is a predictor of platinum sensitivity in sporadic epithelial ovarian cancers

**DOI:** 10.1186/s43556-020-00023-y

**Published:** 2020-12-30

**Authors:** Adel Alblihy, Muslim L. Alabdullah, Michael S. Toss, Mashael Algethami, Nigel P. Mongan, Emad A. Rakha, Srinivasan Madhusudan

**Affiliations:** 1 Translational Oncology, Division of Cancer & Stem Cells, School of Medicine, University of Nottingham Biodiscovery Institute, Nottingham, NG51PB UK; 2 Academic Pathology, Division of Cancer & Stem Cells, School of Medicine, University of Nottingham Biodiscovery Institute, Nottingham, NG51PB UK; 3 Faculty of medicine and Health Sciences, Centre for Cancer Sciences, University of Nottingham, Sutton Bonington Campus, Sutton Bonington, Leicestershire, LE12 5RD UK; 4 Department of Pharmacology, Weill Cornell Medicine, New York, NY 10065 USA; 5 Department of Oncology, Nottingham University Hospitals, Nottingham, NG51PB UK; 6 Division of Cancer & Stem Cells, School of Medicine, Nottingham Biodiscovery Institute, University of Nottingham, University Park, Nottingham, NG7 3RD UK

**Keywords:** DNA repair, RAD50, Ovarian cancer, Predictive biomarker, Platinum therapy

## Abstract

Intrinsic or acquired resistance seriously limits the use of platinating agents in advanced epithelial ovarian cancers. Increased DNA repair capacity is a key route to platinum resistance. RAD50 is a critical component of the MRN complex, a ‘first responder’ to DNA damage and essential for the repair of DSBs and stalled replication forks. We hypothesised a role for RAD50 in ovarian cancer pathogenesis and therapeutics. Clinicopathological significance of RAD50 expression was evaluated in clinical cohorts of ovarian cancer at the protein level (*n* = 331) and at the transcriptomic level (*n* = 1259). Sub-cellular localization of RAD50 at baseline and following cisplatin therapy was tested in platinum resistant (A2780cis, PEO4) and sensitive (A2780, PEO1) ovarian cancer cells. RAD50 was depleted and cisplatin sensitivity was investigated in A2780cis and PEO4 cells. RAD50 deficiency was associated with better progression free survival (PFS) at the protein (*p* = 0.006) and transcriptomic level (*p* < 0.001). Basal level of RAD50 was higher in platinum resistant cells. Following cisplatin treatment, increased nuclear localization of RAD50 was evident in A2780cis and PEO4 compared to A2780 and PEO1 cells. RAD50 depletion using siRNAs in A2780cis and PEO4 cells increased cisplatin cytotoxicity, which was associated with accumulation of DSBs, S-phase cell cycle arrest and increased apoptosis. We provide evidence that RAD50 deficiency is a predictor of platinum sensitivity. RAD50 expression-based stratification and personalization could be viable clinical strategy in ovarian cancers.

## Introduction

Platinating agents such as cisplatin and carboplatin are commonly used in the treatment of ovarian cancer. However not all patients respond and the development of intrinsic or acquired resistance to platinum is a formidable clinical problem in ovarian cancers [1]. Platinum compounds form intra-strand and inter-strand adducts, which if not repaired through the nucleotide excision repair (NER) pathway [2, 3], can contribute to replication arrest leading to double strand breaks (DSB) accumulation [4, 5]. DSBs are detected through the DNA damage signalling and response (DDR) mechanisms which coordinate cell cycle response and DNA repair. Increased DNA repair capacity promotes resistance to platinating agents [6, 7]. On the other hand, reduced DNA repair capacity, such as due to BRCA germ-line deficiency, increase platinum sensitivity. Importantly, PARP inhibitors (Niraparib, Rucaparib, Olaparaib, Talazoparib) are selectively toxic in platinum sensitive BRCA germ line deficient or sporadic epithelial ovarian cancers. Therefore the development of biomarkers that predict platinum sensitivity is an area of unmet clinical need [8].

The MRE11-RAD50-NBS1 (MRN) complex is a ‘first responder’ to DNA damage and is essential for the repair of DSBs and stalled replication forks [9]. RAD50 is a core protein of MRN complex. RAD50 plays a critical role in non-homologous and joining (NHEJ) and homologous recombination (HR) and telomere maintenance [10, 11]. RAD50 is an ATP-modulated DNA cross-linker that has three vital domains: ATP-binding cassette (ABC), zink hook region and MRE11 interaction site [12], and the integrity of these domains is crucial for the process of DSBs by the MRN complex [13]. RAD50 acts as a bridge at the junction of DNA damage, facilitating the recognition and processing of DNA ends by the exonuclease activity of MRE11 to initiate DNA repair. MRE11 endo- and exo- nuclease activities are stimulated by RAD50 [14]. RAD50 deficiency reduces MRE11 nuclear localization and it is interaction with NBS1. RAD50- deficient cells have impaired cell cycle checkpoints and DNA repair capacity [15]. A role for RAD50 in the maintenance of telomere has been described [16]. Germ-line mutations in *RAD50* has been linked to hereditary breast cancers [17]. *RAD50* polymorphisms are associated with increased risk of breast and ovarian cancer [18].

We hypothesised a role for RAD50 in ovarian cancer pathogenesis and therapeutics. In the current study we provide evidence that RAD50 deficiency is a predictor of platinum sensitivity in epithelial ovarian cancers.

## Results

### RAD50 overexpression is linked to aggressive epithelial ovarian cancers

**Fig. 1:**
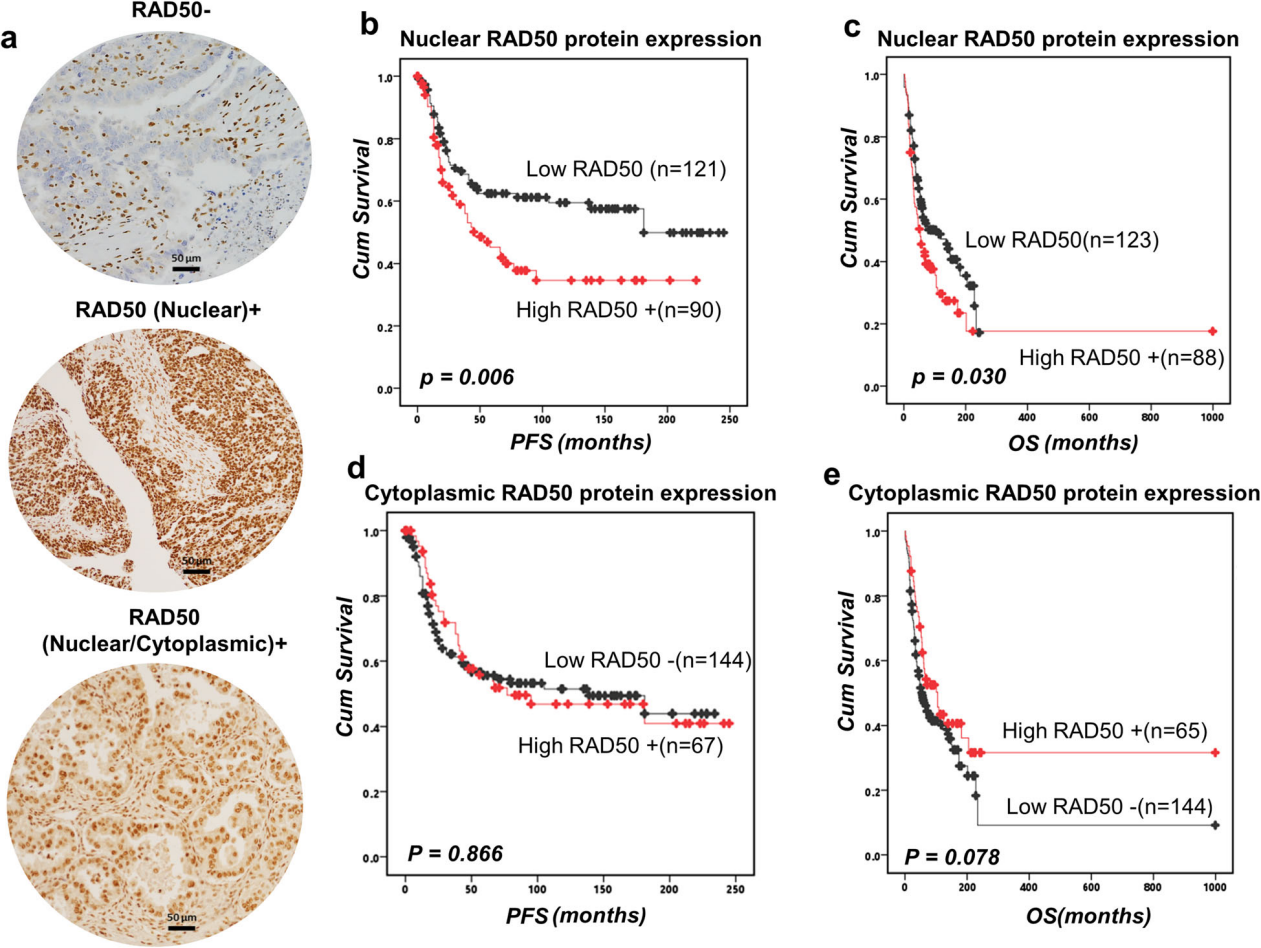
RAD50 protein expression and survival in ovarian cancers. **a** Immunohistochemical expression of RAD50 in ovarian cancers [‘-‘= no tumour expression, ‘+’ = tumour expression]. **b** Kaplan-Meier curve for RAD50 nuclear protein expression and progression free survival (PFS) in ovarian cancer. **c** RAD50 nuclear protein expression and overall survival (OS) in ovarian cancer. **d** Kaplan-Meier curve for RAD50 cytoplasmic protein expression and PFS in ovarian cancer. **e** RAD50 cytoplasmic protein expression and OS in ovarian cancer

Clinicopathological significance of RAD50 protein was evaluated by immunohistochemistry in 331 human ovarian cancers (Fig. 1a). Patient demographics are summarized in Supplementary Table 1. RAD50 protein expression was evaluable in 239 tumours. Negative staining, nuclear staining only or nuclear & cytoplasmic staining was observed in tumours. Intensities of subcellular compartments were each assessed and grouped as follows: 0 = no staining, 1 = weak staining, 2 = moderate staining, 3 = strong staining. The percentage of tumour cells in each category was estimated (0–100%). Histochemical score (H-score) (range 0–300) was calculated by multiplying the intensity of staining and the percentage of staining. A median H-score of ≤120 and 0 was used as the cut-off for low RAD50 nuclear and cytoplasmic expression respectively. Full methodology is described in supplementary methods. We correlated nuclear expression and cytoplasmic expression to clinicopathological outcomes and survival. High nuclear RAD50 was seen in 90/239 (42.6%) tumours and linked to serous cystadenocarcinoma (*p* = 0.033), high grade 3 tumours (*p* = 0.004) (Supplementary Table 2). Low RAD50 expression was associated with better progression free survival (PFS) (*p* = 0.006) (Fig. 1b) and better overall survival (OS) (*p* = 0.003) (Fig. 1c). Cytoplasmic expression of RAD50 did not influence clinicopathological features or survival (Fig. 1d, e, Supplementary Table 2).

In a multivariate model, high nuclear RAD50 (*p* = 0.003) was independently associated with PFS. FIGO stage and platinum sensitivity (*p* < 0.000) were also additional factors independently associated with PFS (Table 1). High nuclear RAD50 (*p* = 0.011), high cytoplasmic RAD50 (*p* = 0.034) along with FIGO stage and platinum sensitivity (*p* < 0.0001) were independent factors associated with OS (Table 1).

**Table 1:** Multivariate analysis

Factors	Progression free survivals (PFS)		Overall survivals (OS)	
	Exp(95% CI)	*P* value	Exp(95% CI)	*P* value
RAD50_N	1.910 (1.242, 2.938)	**0.003**	1.617 (1.117, 2.340)	**0.011**
RAD50_C	0.874 (0.550, 1.388)	0.568	0.637 (0.419, 1.077)	**0.034**
Surgical Pathology Type	0.969 (0.827, 1.136)	0.699	0.930 (0.802, 2.418)	0.332
Surgical Pathology Stage	2.154 (1.651, 2.812)	**0.000**	1.935 (1.549, 2418)	**0.000**
Platinum sensitivity	20.364 (8.642, 47.988)	**0.000**	8.412 (4.532, 15.614)	**0.000**

**Fig. 2:**
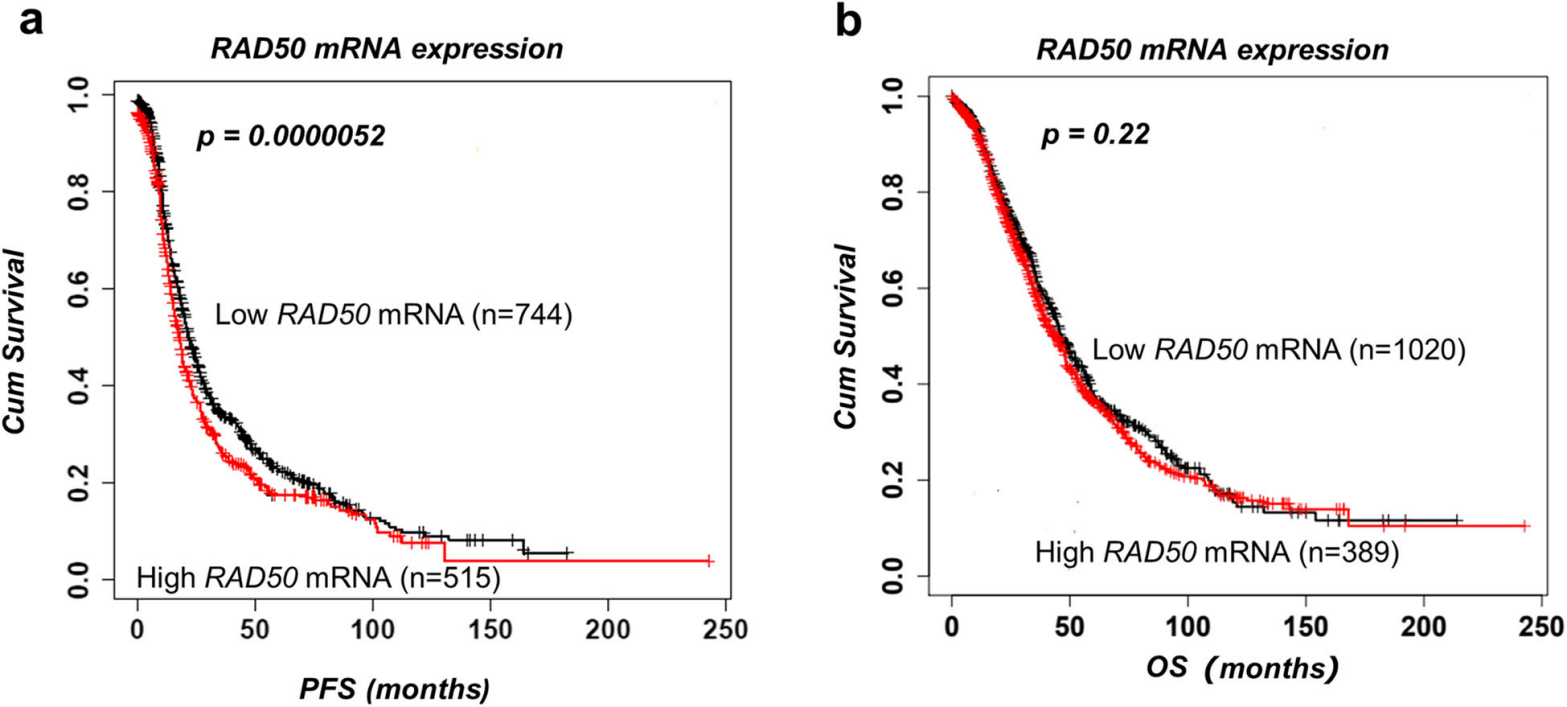
*RAD50* mRNA expression and survival in ovarian cancers. **a** The association between *RAD50* mRNA expression and PFS. **b** The association between *RAD50* mRNA expression and OS

For further validation, we investigated RAD50 mRNA expression in a publicly available online gene expression database of 1259 ovarian cancer cases treated with platinum therapy. High mRNA expression of RAD50 significantly associated with poor PFS (*p* = 0.001) (Fig. 2a) but did not influence OS (*p* = 0.22) (Fig. 2b).

Taken together, the clinical data suggests that RAD50 overexpression could be a predictor of response to platinum therapy. To confirm this hypothesis, we proceeded to pre-clinical studies in platinum sensitive and resistant ovarian cancer cell lines.

### RAD50 level at baseline and following cisplatin therapy in ovarian cancer cells

**Fig. 3:**
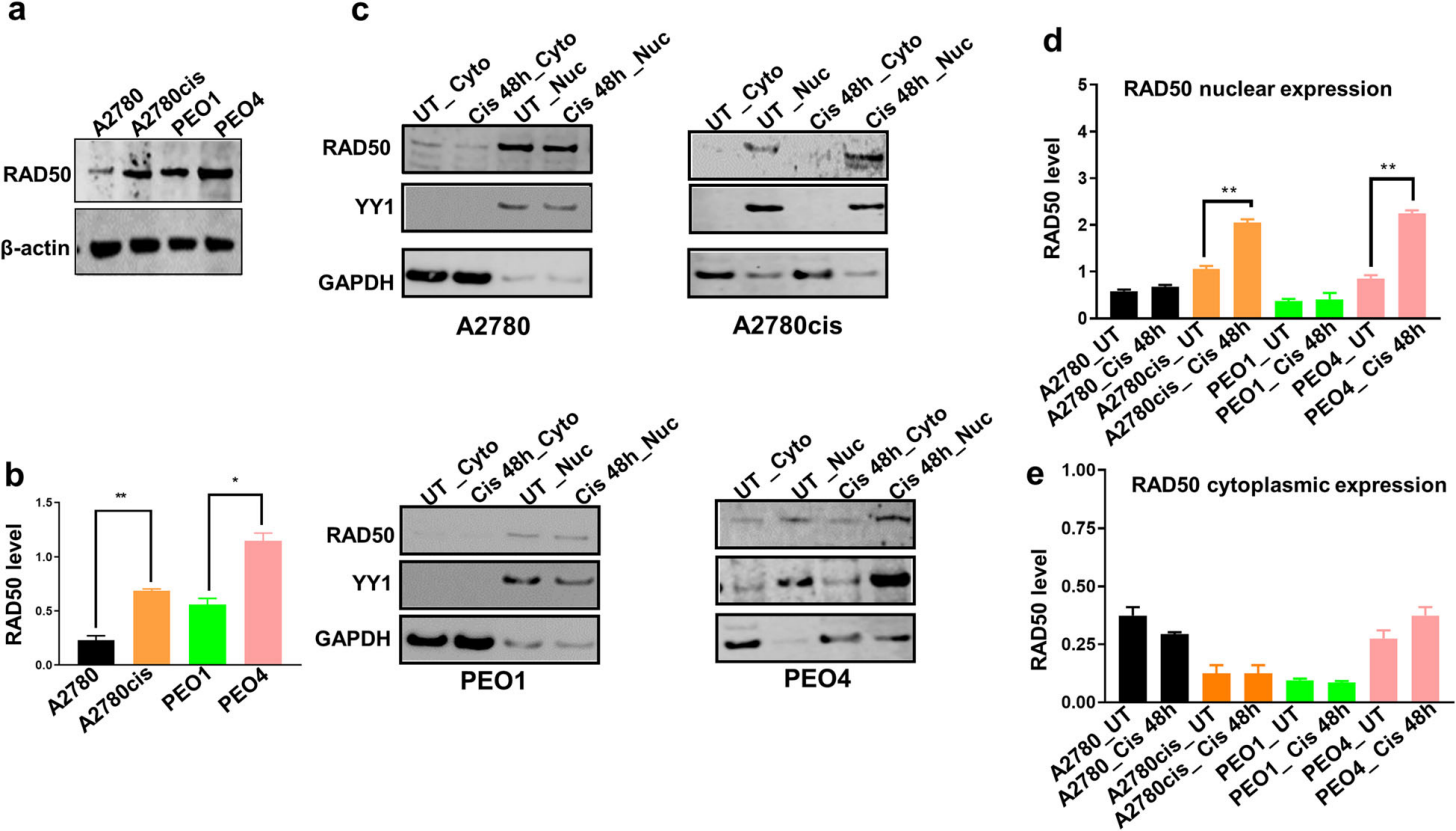
RAD50 protein expression in ovarian cancer cells. **a** Basal RAD50 protein level in A2780, A2780cis, PEO1 and PEO4 cell lines. **b** Quantification of RAD50 baseline levels in A2780, A2780cis, PEO1 and PEO4 cell lines. **c** Western blot of RAD50 protein level in nuclear (Nuc) and cytoplasmic extracts (Cyto) of A2780, A2780cis, PEO1 and PEO4 treated with 5 μM cisplatin. Nuclear and cytoplasmic lysates collected 48 h post treatment. **d** Quantification of RAD50 nuclear sub-cellular localization in A2780, A2780cis, PEO1 and PEO4 cell lines. **e** Quantification of RAD50 cytoplasmic expression in A2780, A2780cis, PEO1 and PEO4 cell lines. YY1 was used as a loading control to the nuclear fractions and GADPH as a loading control for the cytoplasmic fractions. UN = untreated cells. Cis = cisplatin

A2780 cell line is platinum sensitive established from a patient with untreated ovarian cancer. A2780cis cell line is a platinum resistant ovarian cancer developed by continuous exposure of the A2780 cell line to increasing doses of cisplatin. PEO1 platinum sensitive (BRCA2-deficenent) cell line is derived from a patient with a poorly differentiated serous adenocarcinoma treated with platinum-based drugs. PEO4 platinum resistant (BRCA2-proficient) cell line was derived from a malignant effusion from the peritoneal ascites of the same patient after the development of clinical resistance to platinum treatment. The baseline level of RAD50 was investigated in A2780, A2780cis, PEO1 and PEO4 cells. In whole cell lysates, as shown in Fig. 3a, baseline RAD50 protein level was high in A2780cis compared to A2780 cells. Similarly, baseline RAD50 protein level was high in PEO4 compared to PEO1 cells. The quantification of RAD50 baseline levels are shown in Fig. 3b. We then generated nuclear and cytoplasmic extracts at baseline and following 48 h cisplatin therapy. In platinum resistant A2780cis and PEO4 cells, platinum treatment increased RAD50 nuclear sub-cellular localisation compared to platinum sensitive A2780 and PEO1 cells (Fig. 3c). The quantification of nuclear expression of RAD50 is shown in Fig. 3d. No significant alterations were observed for cytoplasmic expression of RAD50 in A2780, A2780cis, PEO1 and PEO4 cells (Fig. 3e). The data suggests that RAD50 protein expression is subjected to sub-cellular localisation upon cisplatin treatment in A2780cis and PEO4 cells.

### RAD50 variant profiling in A2780, A2780cis, PEO1 and PEO4 cells

Germ-line mutations in *RAD50* has been linked to breast and ovarian cancer susceptibility [17]. *RAD50* polymorphisms are associated with increased risk of breast and ovarian cancer [18]. We performed targeted deep sequencing for *RAD50* variants in A2780, A2780cis, PEO1 and PEO4 cells. Ensembl VEP was used to analyse the effect and location of variants using the HG19/GRCh37 genome version. In the parental A2780 line two unique variants were identified (A: 5:131893147-131893147, a novel variant predicted to alter splicing; B: 5:131977963-131977963, rs1804670, a synonymous variant). The Platinum resistant A2780cis harbours a novel unique variant at 5:131973821-131973821 which is predicted to introduce Ala→Asp amino acid substitution. This variant is located within the ATPase domain of RAD50. While the Ala→Asp substitution is similar in size and volume, the introduction of an acidic aspartic acid may influence substrate access to the ATPase domain. Other mutations in this RAD50 domain are found in colon and stomach cancers [19]. Both PE01 and PE04 both harbour a single known variant (5:131892357-131892357, rs2706335) located upstream of the *RAD50* gene which does not alter the coding sequence. We also assessed putative components of the RAD50 interactome (MRE11A, NBN, RINT1, C15ORF26, CELA2B, EP300, GEMIN2, RBBP8, ZFAND2B, ZNF511, RECQL5, MDC1, BARD1, BRCA1, PAXIP1, TERF2, TERF2IP, BLM, DYNLL1, FAM219A, FGFR1OP, H2AFX, ILF2, LRRC39, MAF1, MDM2, PAXIP1, PPARG, TERF2IP, TP53BP1, USP7) for which more than two supporting references were reported in the BioGrid database [20]. Variants were identified in *MRE11A, NBN, RINT1, C15ORF26, CELA2B, EP300, GEMIN2, RBBP8, ZFAND2B, ZNF511, RECQL5* in A2780cis and in *RECQL5, MDC1* in PE04 (Supplementary Table 3). Taken together, the data provides evidence for a role for variants in RAD50 and its associated complex in platinum resistance in ovarian cancer cell lines.

### RAD50 depletion increases cisplatin cytotoxicity in A2780cis and PEO4 cells

**Fig. 4:**
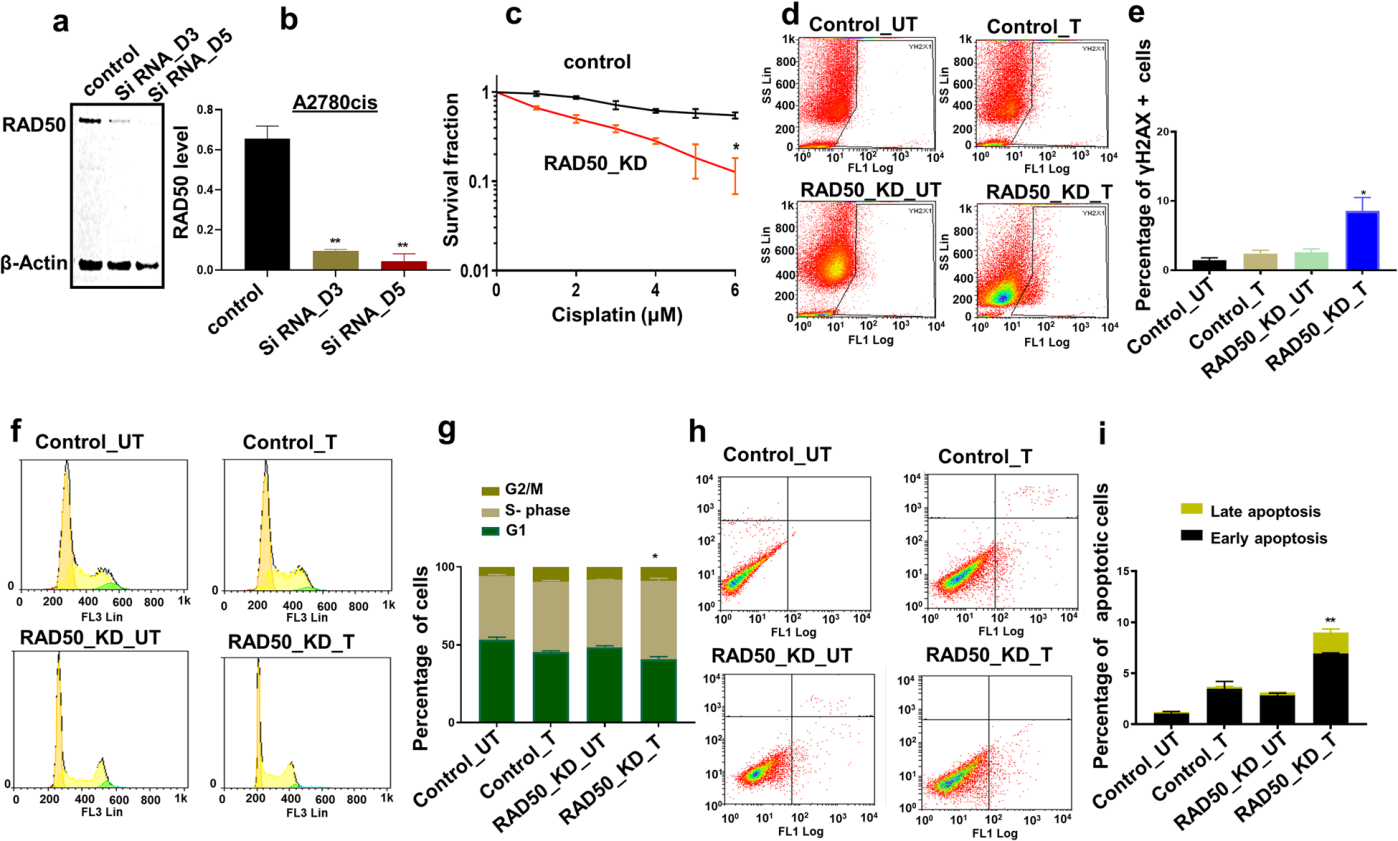
RAD50 depletion and cisplatin sensitivity in A2780cis cells. **a** RAD50_KD in A2780cis cells. **b** Quantification of western blot showing RAD50 depletion in A2780cis cells. **c** Cisplatin sensitivity in A2780cis control and A2780cis_RAD50_KD cells (clonogenic assay). **d** Representative photomicrograph of yH2AX flow cytometry assay for DSBs in control and RAD50_KD_A2780cis cells treated with cisplatin compared to scrambled controls. **e** The percentage of γH2Ax positive cells by flow cytometry in control and RAD50_KD_A2780cis cells treated with cisplatin compared to scrambled controls. **f** Representative photomicrograph of PI flow cytometry assay for cell cycle progression in control and RAD50_KD_A2780cis cells treated with cisplatin compared to scrambled controls. **g** Cell cycle analysis by flow cytometry in control and RAD50_KD_A2780cis cells treated with cisplatin compared to scrambled controls. **h** Representative photomicrograph of Annexin-V flow cytometry assay for apoptotic cells in control and RAD50_KD_A2780cis cells treated with cisplatin compared to scrambled controls. **i** Annexin V analysis by flow cytometry in control and RAD50_KD_A2780cis cells treated with cisplatin compared to scrambled controls. UN = untreated cells. T = treated cells

**Fig. 5:**
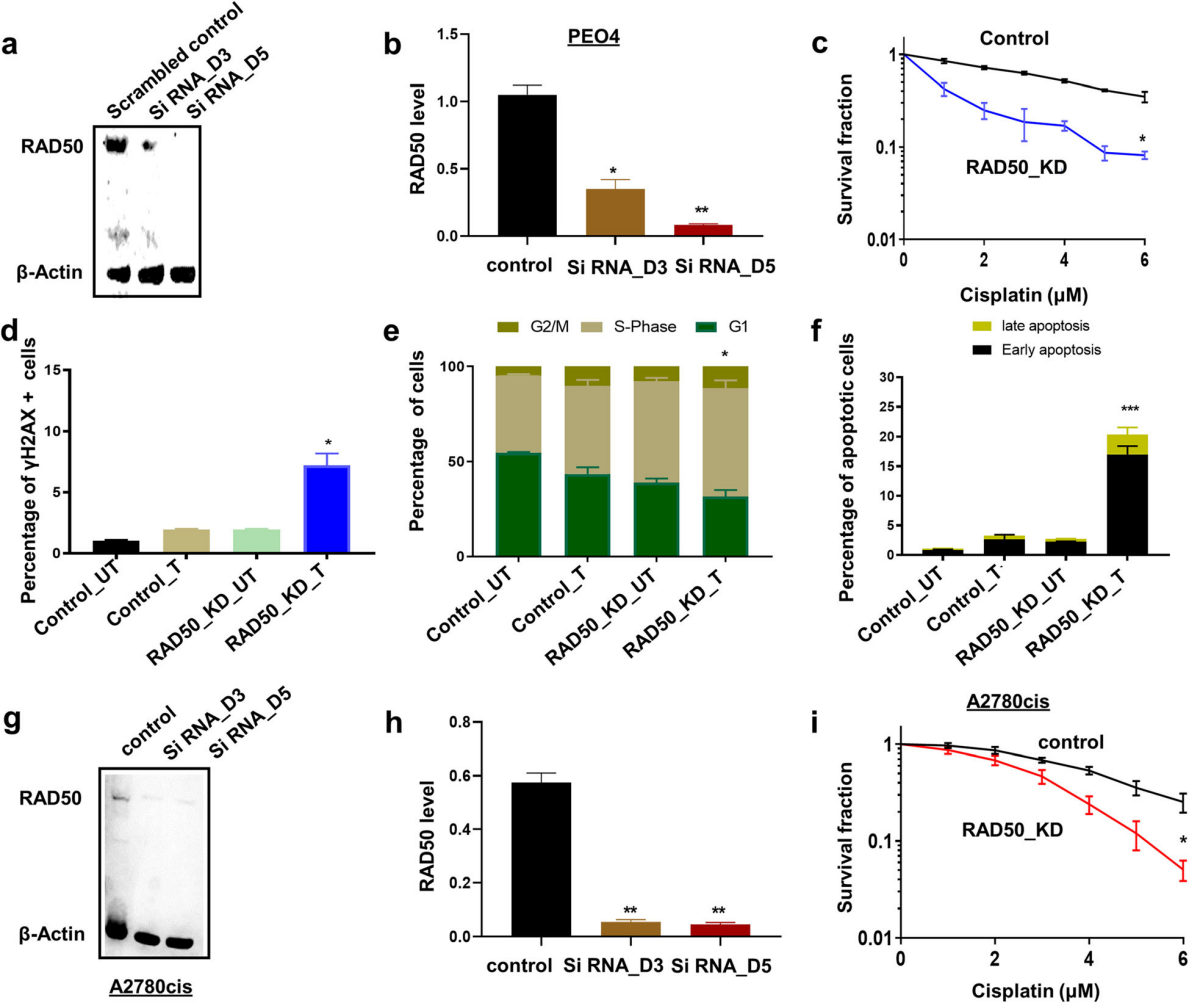
RAD50 depletion and cisplatin sensitivity in PEO4 cells. **a** RAD50_KD in PEO4 cells. **b** Quantification of western blot showing RAD50 depletion in PEO4 cells. **c** Cisplatin sensitivity in A2780cis control and PEO4_RAD50_KD cells (clonogenic assay). **d** The percentage of γH2Ax positive cells by flow cytometry in control and RAD50_KD_ PEO4 cells treated with cisplatin compared to scrambled controls. **e** Cell cycle analysis by flow cytometry in control and RAD50_KD_ PEO4 cells treated with cisplatin compared to scrambled controls. **f** Annexin V analysis by flow cytometry in control and RAD50_KD_ PEO4 cells treated with cisplatin compared to scrambled controls. UN = untreated cells. T = treated cells. **g** RAD50_KD in A2780cis cells using second construct. **h** Quantification of western blot showing RAD50 depletion in A2780cis cells. **i** Cisplatin sensitivity in A2780cis control and A2780cis_RAD50_KD cells (clonogenic assay) using second construct. UN = untreated cells. T = treated cells

We transiently depleted RAD50 using siRNAs in A2780cis cells (Fig. 4a). The Quantification of western blot showing RAD50 depletion in A2780cis cells is demonstrated in Fig. 4b. As shown in clonogenic assays (Fig. 4c), RAD50_knock down (KD)_A2780cis cells were significantly sensitive to cisplatin treatment compared to scrambled controls. Increased sensitivity was associated with DSB accumulation (Fig. 4d, e), S-phase cell cycle arrest (Fig. 4f, g) and increased apoptosis (Fig. 4h, i) compared to scrambled controls. For further validation, we RAD50 using siRNAs in platinum resistant PEO4 cells (Fig. 5a). The Quantification of western blot showing RAD50 depletion in PEO4 cells is demonstrated in Fig. 5b. As expected, RAD50_KD_PEO4 cells (Fig. 5c) showed increased platinum sensitivity which was associated with DSB accumulation (Fig. 5d), S-Phase arrest (Fig. 5e) and apoptotic cells (Fig. 5f). For additional validation, we tested another siRNA construct for RAD50 depletion in A2780cis cells (Fig.5g). The Quantification of western blot showing RAD50 depletion in A2780cis cells is shown in Fig. 5h. As expected, RAD50 depletion lead to platinum sensitization compared to scrambled controls (Fig. 5i).

Taken together, the clinical and pre-clinical data provides evidence that RAD50 is a predictor of platinum sensitivity in ovarian cancer.

## Discussion

RAD50 has critical roles during HR, NHEJ and telomere maintenance [13]. Here we provide evidence that RAD50 expression is a predictor of platinum sensitivity. High RAD50 expression was associated with aggressive high-grade serous cystadenocarcinomas. In contrast, low RAD50 expression was observed in low-grade epithelial ovarian cancer in another study [21]. At the protein and transcriptomic level, we show that high RAD50 expression was associated with poor PFS in patients. Our data would concur with a previous study in resected non-small-cell lung cancer (NSCLC) where high RAD50 expression was associated with poor survival following radiotherapy [10]. In gastric cancer [22], colorectal cancer [23] and rectal cancers [24], similarly, high RAD50 has been associated with poor clinical outcomes in patients. In the current study cytoplasmic expression of RAD50 was not significantly associated with progression free survival or overall survival. There was a non-significant trend of better survival in tumours with high cytoplasmic expression of RAD50. We speculate that cytoplasmic sequestration of DNA repair protein such as RAD50 could result in reduced nuclear levels resulting in improved survival in patients after cisplatin-based chemotherapy. In ovarian cancer cell lines, following cisplatin therapy, we observed increased nuclear accumulation of RAD50 protein in nuclear extracts in platinum resistant cell lines compared to platinum sensitive cell lines. However, a limitation is that we did not validate this observation using confocal microscopy. Nevertheless, RAD50 depletion in platinum resistant ovarian cancer cells increased cisplatin cytotoxicity. Increased toxicity was associated with DSB accumulation as evidenced In a head and neck squamous cell carcinoma model, RAD50 blockade resulted in cisplatin chemosensitization [13]. Furthermore, RAD50 depletion also sensitized human breast cancer cells to cisplatin treatment [18]. Disrupting RAD50 function has been shown to sensitize human nasopharyngeal carcinoma cells to radiotherapy [12]. In NSCLC cells, RAD50 depletion not only increased radiation-sensitivity but RAD50 overexpression enhanced radio-resistance in vitro [10]. The data would suggest that RAD50 is a predictor of response to DNA damaging cytotoxic therapy.

Germ-line mutations in *RAD50* has been linked to breast and ovarian cancer susceptibility [17]. *RAD50* polymorphisms are also associated with increased risk of breast and ovarian cancer [18]. In ovarian cancer cell lines, however, RAD50 variants have not been described previously. Therefore, we conducted a deep sequencing study and identified a unique RAD50 variant at 5:131973821-131973821 in A2780cis cells which is predicted to alter RAD50 function. However, detailed mechanistic study would be required to evaluate whether the variation contributes to platinum resistance. In addition, as RAD50 is multi-functional protein with previously reported interacting partners, we conducted a bioinformatic analyses to understand RAD50 interactome in ovarian cancer cells. The interactome revealed several partners (including key DNA repair genes such as NBS1, Mre11) with roles in platinum resistance. Together, the data provides evidence that RAD50 directly and indirectly (through its interactors) influence response to platinum induced DNA damage in cells.

BRCA1 interacts with RAD50 [25, 26] during DSB repair. In addition, during early stages of DSB repair via HR, DSBs activate ATM and ATR kinases which in turn phosphorylate p53 and BRCA1. During subsequent stages of HR, MRN resects the DNA to form 3″ overhangs. This is followed by loading of RAD51 onto RPA-coated DNA under the influence of BRCA2 [26]. BRCA1 germ-line deficiency or RAD50 inactivation results in defective HR. Interestingly, a link between RAD50 deficiency, BRCAness phenotype and PARP inhibitor sensitivity has been shown in ovarian cancers [27]. In *BRCA* wild-type ovarian cancers, *RAD50* deletion was shown in 18% of tumours and correlated better PFS and OS. RAD50 depletion in ovarian cancer cell lines also increased response to PARP inhibitor therapy [27]. The data including ours provide evidence that RAD50 deficiency is not only a marker of platinum sensitivity but could also predict response to PARP inhibitor therapy in epithelial ovarian cancers.

## Materials and methods

### Clinical study

#### RAD50 expression level in ovarian cancers

Evaluation of the expression of RAD50 was performed on tissue microarrays of 331 consecutive sporadic epithelial ovarian cancer cases treated at Nottingham University Hospitals (NUH) between 1997 and 2010. This study was carried out in accordance with the declaration of The Helsinki. The study was approved by the Nottingham Research Biorepository (NRB) Access Committee under the biobank ethical approval REC reference: 10/H1008/72 (NRES Committee North West - Greater Manchester Central). All patients provided informed consent. Patient demographics is summarized in Supplementary Table 1. Detailed methodology for immunohistochemical evaluation of RAD50 expression and statistical analyses is described in supplementary methods.

#### *RAD50* transcript in ovarian cancers

*RAD50* mRNA expression was evaluated in a publically available online gene expression dataset of 1259 ovarian cancer patients treated with platinum-based chemotherapy from 15 previously published studies and available at ‘http://kmplot.com/analysis/index.php?p=service&cancer=ovar’.

### Pre-clinical study

#### Cell lines and tissue culture

A2780 (platinum sensitive) A2780cis (platinum resistant) were purchased from Sigma Aldrich (Gillingham, UK). PEO1 (BRCA2-deficient, platinum sensitive) and PE04 (BRCA2-proficient, platinum resistant) were purchased from American Type Culture Collection (ATCC, Manassas, USA). Cells cultured in RPMI (R8758, Merck, UK) supplemented with 10% FBS (F4135, Merck, UK), 1% Penicillin-Streptomycin (P4333, Merck, UK). All cell lines were maintained in a humidified incubator at 37 °C in a 5% CO2 atmosphere.

#### Western blot analysis

Cells were trypsinized and lysed in RIPA buffer (R0278, Sigma.UK) with the addition of protease cocktail inhibitor (P8348, Sigma, UK), phosphatase inhibitor cocktail 2 (P5726, Sigma, UK) and phosphatase inhibitor cocktail 3 (P0044, Sigma) and stored at − 20 °C. Protein quantification was performed using BCA Protein Assay kit (23225, Thermofisher, UK). Samples were run on SDS-bolt gel (4–12%) bis-tris. Membranes were then incubated with primary antibodies as follows: RAD50 (1:500, ab89), ß-actin (1:1000, ab8226), YY1 (1:1000, ab109228), GADPH (1:1000, ab9485). Membranes were then washed and incubated with Infrared dye-labelled secondary antibodies (LiCor) [IRDye 800CW Donkey Anti-Rabbit IgG (926-32213) and IRDye 680CW Donkey Anti-Mouse IgG (926-68072)] at dilution of 1:10,000 for 1 h. Membranes were scanned with a LiCor Odyssey machine (700 and 800 nm) to determine protein levels.

#### Nuclear and cytoplasmic extracts

Cells were seeded in T25 flasks overnight. Cells were then treated with 5 μM of cisplatin and left at 37 °C in a 5% CO2 atmosphere for 48 h. Cells were then harvest by trypsinization, washed with PBS and centrifuged at 1000×g for 5 min. Cell Lysates were extracted using the NE-PER Nuclear and Cytoplasmic Extraction Reagents (78833, Thermofisher, UK). Cells were collected by trypsinization, washed with PBS and centrifuged at 1000×g for 5 min. Extracts were quantified using BCA protein quantification kit (Thermo Fisher Scientific) and protein levels were checked by western blot. YY1 was used as a loading control to the nuclear fractions and GADPH as a loading control for the cytoplasmic fractions.

#### Transient knockdowns of RAD50

RAD50 (ID S792) and the validation construct of RAD50 (ID S793) siRNAs oligonucleotides were purchased from Invitrogen. Lipofectamine 3000 reagent (L3000015, Invitrogen, UK) was used according to the manufacturer’s protocol. Briefly cells were seeded at 50–60% confluency in T25 flasks overnight. Cells were transfected with 20 nM of siRNA oligonuclotide or scrambled SiRNA oligonucleotide control (4390843, Thermofiher) in Opti-MEM media (31985-062, Gibco).

#### Clonogenic assays

In the clonogenic assay, 32 cells/cm^2^ were seeded in 6-well plates and left at 37 °C in a 5% CO_2_ atmosphere. Cisplatin (Kindly provided by Nottingham University Hospital) was added at the indicated concentrations and the plates were left at 37 °C in a 5% CO2 atmosphere for 14 days. Later the plates were washed with PBS, fixed and stained and colonies were counted. Survival fraction (SF) were calculated using the formula SF = no. of colonies formed after treatment/no. of cells seeded x platting efficiency. Number of colonies counted were normalised relative to the count of untreated wells which were considered as 100% survival.

#### Cell cycle and apoptosis by flow Cytometry

1 × 10^5^ Cells per well were seeded in 6-well plates overnight. Cells were treated with Cisplatin (1 μM) for A2780 cells and (5 μM) for A2780 cis cells. After 24 h Cells were trypsinized and washed with ice cold PBS, then fixed in 70% ethanol for at least 30 mins. After removal of the fixative solution by centrifugation cells were stained with phospho Histone (γH2AX) Ser139. Cells were then treated with RNase and DNA content were stained with 10μg/ml propidium iodide (Sigma Aldrich) in PBS. For Apoptosis detection, cells were collected by trypsinization after 24 h washed and analysed using annexinV detection kit (BD biosciences). Samples were analysed on FC500 flow cytometer (Beckman Coulter) and data were analysed using Weasel software. Data were generated using GraphPad Prism7 software.

#### Statistical analysis

Data was conducted as on GraphPad Prism 7 software. To compare between two groups, student- T-tests analysis was performed. One-way ANOVA was performed to compare between more than two groups (variances analyses). Two-way ANOVA was used to analyse two variables such as Annexin V analysis and cell cycle analysis. All experiments were expressed as means ± standard deviation S.D. of three independent experiments. *p*-values < 0.05 = *, *p*-value < 0.01 = ** & *p*-value < 0.001 = ***.

#### Targeted next generation sequencing and bioinformatics

Genomic DNA was extracted from cell lines using the PicoPure™ DNA Extraction Kit (Thermofisher,UK). Targeted next generation sequencing was used to identify genomic variants in platinum sensitive (A2780) and platinum resistant derivatives (A2780cis). The SureSelect All Exon V5 kit (Agilent Technologies) was used to enrich for protein coding regions and sequencing performed using an Illumina NextSeq500 sequencer with paired end reads (150 bp) and a minimum of 88million reads generated per sample. Raw reads were fastq formatted. Contaminating adapter sequences and low-quality sequences were processed using Skewer [28]. Quality processed reads were aligned to the HG19 reference genome using BWA [29], duplicate alignments identified and processed using PicardTools, and realignment completed using the Abra assembly based realigner [30] to enhance detection of insertion/deletion variants. Variant calling and filtering was completed using Samtools/Bcftools (v1.3.1) [31]. Variants, in variant call format (VCF), associated with Platinum resistance were identified using Vcftools [32]. Variants were annotated and functional significance assessed using the Ensembl Variant Effect Predictor tool [33]. Library preparation and sequencing was conducted by Source Biosciences (Nottingham, UK).

In accordance with the journal’s guidelines, we will provide our data for the reproducibility of this study in other centres if such is requested.

## Supplementary Information

**Additional file 1:** **Supplementary Table 1.** Patient demographics and pathological features in ovarian cancer. **Supplementary Table 2**. The correlation between RAD50 nuclear and cytoplasmic expression and clinicopathological parameters. **Supplementary Table 3.** Cell cycle quantification following cisplatin treatment in control and RAD50_KD cell lines. **Supplementary methods**.

## Data Availability

All data and material relevant to this publication is available upon reasonable request.

## Abbreviations

ABC: ATP-binding cassette
DDR: DNA damage signalling and response
DSB: Double strand break
HR: Homologues recombination
MRN: The MRE11-RAD50-NBS1
NER: Nucleotide excision repair
NHEJ: Non-homologous and joining
OS: Overall survivals
PFS: Progression free survivals
