# Supplementary material for: RAD50 deficiency is a predictor of platinum sensitivity in sporadic epithelial ovarian cancers
Source: Mol Biomed. 2020 Dec 30;1:19. doi: 10.1186/s43556-020-00023-y (PMC8607373; doi:10.1186/s43556-020-00023-y)
Supplement: Supplementary file 1 — Additional file 1: Supplementary Table 1. Patient demographics and pathological features in ovarian cancer. Supplementary Table 2. The correlation between RAD50 nuclear and cytoplasmic expression and clinicopathological parameters. Supplementary Table 3. Cell cycle quantification following cisplatin treatment in control and RAD50_KD cell lines. Supplementary methods. [file 43556_2020_23_MOESM1_ESM.zip › Supplementary methods and tables.docx]

**SUPPLEMENTARY METHODS**

**Tissue microarray (TMA) and immunohistochemistry (IHC):** Tumours were arrayed in tissue microarrays (TMAs) constructed with 2 replicate 0.6mm cores from the tumours. Immunohistochemical staining was conducted using the Thermo Fisher Scientific Shandon Sequenza chamber system (REF: 72110017), in combination with the Novolink Max Polymer Detection System (RE7280-K: 1250 tests), and the Leica Bond Primary Antibody Diluent (AR9352), each used according to the manufacturer’s instructions (Leica Microsystems). The tissue slides were deparaffinised with xylene and then rehydrated through five decreasing concentrations of alcohol (100%, 90%, 70%, 50% and 30%) for two minutes each. Pre-treatment antigen retrieval was performed on the TMA sections using sodium citrate buffer (pH 6.0) and heated for 20 minutes at 95^°^C in a microwave (Whirlpool JT359 Jet Chef 1000W). A set of slides were incubated with the primary anti-RAD50 mouse monoclonal antibody (clone ab89, Abcam), at a dilution of 1:400, for 1 hour at room temperature. Negative (by omission of the primary antibody and IgG-matched serum) and positive controls were included in each run.

**Evaluation of immune staining:** Whole field inspection of the core was scored, and the subcellular localisation of each marker was identified (nuclear, cytoplasm, cell membrane). Intensities of subcellular compartments were each assessed and grouped as follows: 0 = no staining, 1 = weak staining, 2 = moderate staining, 3 = strong staining. The percentage of tumour cells in each category was estimated (0–100%). Histochemical score (H-score) (range 0–300) was calculated by multiplying the intensity of staining and the percentage of staining. A median H-score of ≤120 and 0 was used as the cut-off for low RAD50 nuclear and cytoplasmic expression respectively. Not all cores within the TMA were suitable for IHC analysis due to missing cores or absence of tumour cells.

**Statistical analysis:** Correlation with clinical and pathological parameters using categorised data was identified using Chi-squared test. All tests were 2-tailed. Survival rates were determined using Kaplan–Meier method and compared by the log-rank test. All analyses were conducted using Statistical Package for the Social Sciences (SPSS, version 22, Chicago, IL, USA) software for windows. P value of less than 0.05 was identified as statistically significant.

| **Characteristics** | | **Number** | **Percentages** |
| --- | --- | --- | --- |
| ***Pathology*** | | | |
|  | Serous cystadenocarcinoma | 178 | 53.9% |
|  | Endometrioid | 44 | 13.2% |
|  | Clear cell carcinoma | 24 | 7.2% |
|  | Mucinous cystadenocarcinoma | 46 | 13.8% |
|  | Others | 15 | 4.5% |
|  | Mixed | 17 | 5.1% |
| ***Grade*** | | | |
|  | 1 | 46 | 13.8% |
|  | 2 | 60 | 18% |
|  | 3 | 171 | 51.5% |
| ***Residual tumour*** | | | |
|  | None/Microscopic | 205 | 61.7% |
|  | <1cm | 34 | 10.2% |
|  | >1-2 cm | 15 | 4.5% |
|  | >2cm | 40 | 12% |
| ***FIGO Stage*** | | | |
|  | I | 123 | 37% |
|  | II | 49 | 14.7% |
|  | III | 128 | 38.5% |
|  | IV | 11 | 3.3% |
| ***Chemotherapy*** | | | |
|  | Carboplatin monotherapy | 81 | 24.3% |
|  | Carboplatin + Paclitaxel | 86 | 25.9% |
| ***Platinum sensitivity*** | | | |
|  | Sensitive | 250 | 75.3% |
|  | Resistant | 26 | 7.8% |
|  | Unknown | 55 | 16.5% |
| ***Relapse status*** | | | |
|  | Progression-free | 169 | 50.9% |
|  | Progressed/relapsed | 118 | 35.5% |
|  | Unknown | 44 | 13.2% |

**Supplementary Table 1:** Patient demographics and pathological features in ovarian cancer.

**Supplementary Table 2**. The correlation between RAD50 nuclear and cytoplasmic expression and clinicopathological parameters.

|  | **RAD50 _cyto –**  **N(%)** | **RAD50_cyto +**  **N(%)** | **P- value** | **RAD50_nuc –**  **N(%)** | **RAD50_nuc +**  **N(%)** | **P- value** |
| --- | --- | --- | --- | --- | --- | --- |
| ***Pathological Type*** |  |  | 0.153 |  |  | ***0.033*** |
| Serous adenocarcinoma | 88 (63.3%) | 51 (36.7%) |  | 64 (46%) | 75 (54%) |  |
| Mucinous adenocarcinoma | 20 (64.5%) | 11 (35.5%) |  | 22 (71) | 9 (29%) |  |
| Endometrioid carcinoma | 23 (74.2%) | 8 (25.8%) |  | 21 (67.7%) | 10 (32.3%) |  |
| Clear cell carcinoma | 17 (94.4%) | 1 (5.6%) |  | 12 (66.7%) | 6 (33.3%) |  |
| Mixed | 8 (66.7%) | 4 (33.3%) |  | 6 (50%) | 6 (50%) |  |
| ***FIGO Stage*** |  |  | 0.433 |  |  | 0.572 |
| I | 62 (70.5%) | 26 (29.5%) |  | 51 (58%) | 37 (42%) |  |
| II | 25 (75.8%) | 8 (24.2%) |  | 15 (45.5%) | 18 (54.5%) |  |
| III | 66 (62.9%) | 39 (37.1%) |  | 59 (59.2%) | 46 (43.8%) |  |
| IV | 4 (57.1%) | 3 (42.9%) |  | 3 (42.9%) | 4 (57.1%) |  |
| ***Tumour Grade*** |  |  | 0.274 |  |  | ***0.004*** |
| Grade 1 | 22 (73.3%) | 8 (26.7%) |  | 24 (80%) | 6 (20%) |  |
| Grade 2 | 33 (73.3%) | 12 (26.7%) |  | 26 (57.8%) | 19 (42.2%) |  |
| Grade 3 | 81 (62.3%) | 44 (37.7%) |  | 61 (46.9%) | 69 (53.1%) |  |
| ***Surgical Optimal Debulking*** |  |  | 0.377 |  |  | 0.134 |
| Optimally Debulked | 120 (68.2%) | 56 (31.8%) |  | 103 (58.5%) | 73 (41.5%) |  |
| Not Optimally Debulked | 27 (64.3%) | 56 (31.8%) |  | 20 (47.6%) | 22 (52.4%) |  |
| Non | 101 (68.7%) | 46 (31.3%) |  | 86 (58.5%) | 61 (41.5%) |  |
| Measurable | 42 (46.6%) | 23 (35.4%) |  | 34 (52.3%) | 31 (47.7%) |  |

**Supplementary Table 4.** Cell cycle quantification following cisplatin treatment in control and RAD50_KD cell lines.

| **Cell line** | **G1**  **(Mean ± SD*)** | **S phase**  **(Mean ± SD*)** | **G2/M**  **(Mean ± SD*)** |
| --- | --- | --- | --- |
| **A2780 cis (C) _UT** | 52.2 ± 1.7 | 40.7 ± 1.06 | 6 ± 0.7 |
| **A2780 cis (C) _Cisplatin** | 45.4 ± 0.7 | 45± 0.8 | 9.5 ± 0.7 |
| **A2780 cis_** **RAD50KD _UT** | 48.6 ± 0.8 | 43 ± 0.2 | 7.58.2 ± 0.6 |
| **A2780 cis _RAD50_KD__Cisplatin** | 40.7 ± 1.7 | 50.2 ±1.7 | 10.2 ± 1 |
|  |  |  |  |
| **PEO4 (C) _UT** | 54.5 ± 0.7 | 41 ± 1.4 | 6.5 ± 0.7 |
| **PEO4 (C) _Cisplatin** | 48.5 ± 2.1 | 44.5 ± 2.1 | 8 ± 0.7 |
| **PEO4_** **RAD50_KD _UT** | 47 ± 1.4 | 45.4 ± 0.7 | 7 ± 0.7 |
| **PEO4 _RAD50_KD__Cisplatin** | 33.5 ± 3.5 | 57 ± 2.8 | 9 ± 0.35 |
